# Low human dystrophin levels prevent cardiac electrophysiological and structural remodelling in a Duchenne mouse model

**DOI:** 10.1038/s41598-021-89208-1

**Published:** 2021-05-07

**Authors:** Gerard A. Marchal, Maaike van Putten, Arie O. Verkerk, Simona Casini, Kayleigh Putker, Shirley C. M. van Amersfoorth, Annemieke Aartsma-Rus, Elisabeth M. Lodder, Carol Ann Remme

**Affiliations:** 1 Department of Experimental Cardiology, Amsterdam UMC (Location AMC), Meibergdreef 9, 1005 AZ Amsterdam, The Netherlands; 2 Department of Human Genetics, Leiden University Medical Center, Albinusdreef 2, 2333 ZA Leiden, The Netherlands; 3 Amsterdam UMC (Location AMC), Department of Medical Biology, Meibergdreef 9, 1005 AZ Amsterdam, The Netherlands

**Keywords:** Cardiovascular diseases, Arrhythmias, Cardiovascular biology, Arrhythmias

## Abstract

Duchenne muscular dystrophy (DMD) is a progressive neuromuscular disorder caused by loss of dystrophin. This lack also affects cardiac structure and function, and cardiovascular complications are a major cause of death in DMD. Newly developed therapies partially restore dystrophin expression. It is unclear whether this will be sufficient to prevent or ameliorate cardiac involvement in DMD. We here establish the cardiac electrophysiological and structural phenotype in young (2–3 months) and aged (6–13 months) dystrophin-deficient *mdx* mice expressing 100% human dystrophin (hDMD), 0% human dystrophin (hDMDdel52-null) or low levels (~ 5%) of human dystrophin (hDMDdel52-low). Compared to hDMD, young and aged hDMDdel52-null mice displayed conduction slowing and repolarisation abnormalities, while only aged hDMDdel52-null mice displayed increased myocardial fibrosis. Moreover, ventricular cardiomyocytes from young hDMDdel52-null animals displayed decreased sodium current and action potential (AP) upstroke velocity, and prolonged AP duration at 20% and 50% of repolarisation. Hence, cardiac electrical remodelling in hDMDdel52-null mice preceded development of structural alterations. In contrast to hDMDdel52-null, hDMDdel52-low mice showed similar electrophysiological and structural characteristics as hDMD, indicating prevention of the cardiac DMD phenotype by low levels of human dystrophin. Our findings are potentially relevant for the development of therapeutic strategies aimed at restoring dystrophin expression in DMD.

## Introduction

Duchenne muscular dystrophy (DMD) is an X-linked genetic neuromuscular disorder affecting around 20 in 100,000 male live births^1,2^. DMD is caused by mutations affecting the open reading frame of the *DMD* gene, resulting in absence or trace levels of functional dystrophin^3,4^. DMD patients develop progressive muscle wasting and weakness during early childhood^5^. While respiratory complications in combination with cardiac dysfunction traditionally led to mortality around the start of adulthood^6^, recent improved access to mechanical ventilation has prolonged survival up to the age of 30, with cardiac dysfunction now emerging as a major cause of death in DMD patients^7^. The majority of DMD patients display electrocardiogram (ECG) abnormalities at a very young age, and virtually all patients have cardiac manifestations at adolescence, including cardiomyopathy, heart failure, myocardial fibrosis, conduction disease, and ventricular arrhythmias^8,9^. Studies in DMD (*mdx*) mouse models have similarly demonstrated that loss of dystrophin leads to cardiac abnormalities, including fibrosis^10–12^ and electrophysiological disruptions^13–17^.

Dystrophin is highly expressed in cardiomyocytes, where it is enriched in costameres and T-tubules at the lateral membrane^18^. Dystrophin modulates several ion channels, including the cardiac sodium channel Na_V_1.5, which is responsible for cardiomyocyte depolarisation by mediating the fast upstroke of the action potential (AP)^19,20^. Na_V_1.5 indirectly interacts with dystrophin via syntrophin and expression levels of the latter are also robustly decreased in DMD^21,22^. In various DMD mouse models it has been demonstrated that loss of dystrophin leads to a decrease in sodium current (*I*_Na_), a known risk factor for arrhythmias and sudden cardiac death^20,23^. Crucially, a combination of *I*_Na_ loss and myocardial fibrosis may result in a markedly disturbed cardiac conduction, greatly increasing the risk for potentially lethal cardiac arrhythmias in DMD patients. Hence, (early) loss of Na_V_1.5 and/or *I*_Na_ is potentially a major contributing factor to the cardiac phenotype in DMD patients.

In contrast to DMD, where there is (virtual) absence of dystrophin due to frameshift mutations, mutations in the *DMD* gene causing Becker muscular dystrophy (BMD) often maintain the reading frame of *DMD*, but result in an internally truncated, though partially functional dystrophin protein^24,25^. The clinical (including cardiac) phenotype of BMD is usually presented at a later age and is generally less progressive than in DMD, with survival only mildly impaired^26,27^. Hence, genetic therapies aimed at restoring dystrophin expression, even only at relatively low levels and/or of partially functional proteins, may constitute a potentially promising approach in DMD. Of these, exon skipping has been approved in Japan and the USA, but this was based on restoration of very low levels (< 1–5%) of Becker-like dystrophins in skeletal muscle biopsies^28,29^. Functional effects have still to be confirmed and it is unclear whether these very low amounts of dystrophin will prevent disease progression in skeletal and cardiac muscles. We here investigated whether low expression levels (~ 5%) of dystrophin are sufficient to prevent detrimental cardiac alterations associated with DMD. In addition to the humanized *mdx* (hDMDdel52-null) mouse model^30^ expressing no functional dystrophin, we used a transgenic mouse dystrophin-negative model expressing normal levels of functional human dystrophin^30,31^, here referred to as hDMD mice. We compared these to hDMDdel52-low mice, which display trace levels (~ 5%) of functional human dystrophin in all myofibers and a mild dystrophic phenotype^30^. Our findings demonstrate that low dystrophin levels are sufficient to prevent the cardiac and structural electrophysiological abnormalities associated with DMD, which is of potential relevance for the development of novel therapeutic strategies aimed at restoring dystrophin expression in DMD.

## Results

### Low dystrophin expression is sufficient to rescue ECG parameters

**Figure 1:**
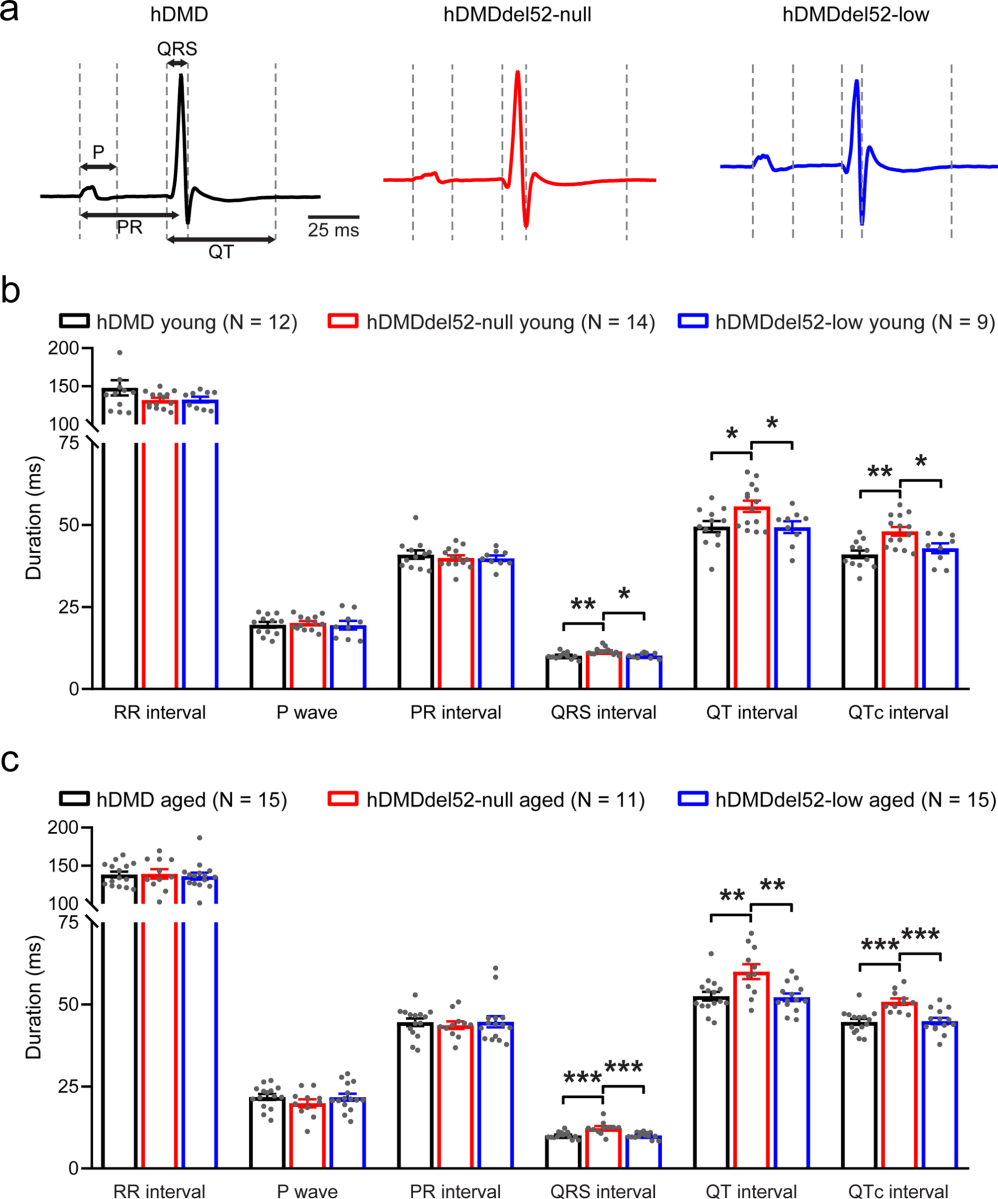
ECG measurements in young and aged mice show ventricular conduction slowing and delayed repolarisation in the absence of dystrophin, prevented by low expression of dystrophin. (**a**) Representative averaged ECG traces obtained in young hDMD (black), hDMDdel52-null (red), and hDMDdel52-low (blue) mice. (**b**) Average ECG characteristics of young hDMD (2.3 ± 0.1 months), hDMDdel52-null (2.4 ± 0.1 months), and hDMDdel52-low mice (2.4 ± 0.1 months). (**c**) Average measurements in aged hDMD (8.6 ± 0.5 months), hDMDdel52-null (10.0 ± 0.5 months), and hDMDdel52-low mice (8.4 ± 0.5 months). N represents the number of animals measured. *P < 0.05, **P < 0.01, ***P < 0.001 (one-way ANOVA with Tukey’s post-hoc test).

To assess the effect of different levels of dystrophin on cardiac electrical activity, we performed ECG measurements on anesthetized young (age 2–3 months) and aged (age 6–13 months) mice (Fig. 1a). Measurements on young hDMD mice expressing 100% functional human dystrophin and young hDMDdel52-null mice lacking dystrophin revealed that absence of functional dystrophin resulted in prolonged QRS duration (reflecting ventricular conduction) and QT(c) interval (a marker of ventricular repolarisation), while RR interval, P wave duration, and PR interval (reflecting heart rate, atrial conduction, and atrio-ventricular conduction, respectively) remained unaffected (Fig. 1b). ECG assessment in young hDMDdel52-low mice showed that QRS and QT(c) prolongation were prevented by the presence of low levels (~ 5%) of functional human dystrophin (Fig. 1b). As in young animals, ECG measurements in aged hDMD and hDMDdel52-null mice revealed QRS and QT(c) prolongation in the absence of dystrophin, which was prevented by low dystrophin expression in aged hDMDdel52-low mice (Fig. 1c). Hence, low levels (~ 5%) of human dystrophin are sufficient to prevent the ECG phenotype observed in young and aged DMD mice.

### Low dystrophin expression prevents left ventricular structural abnormalities secondary to dystrophin loss

**Figure 2:**
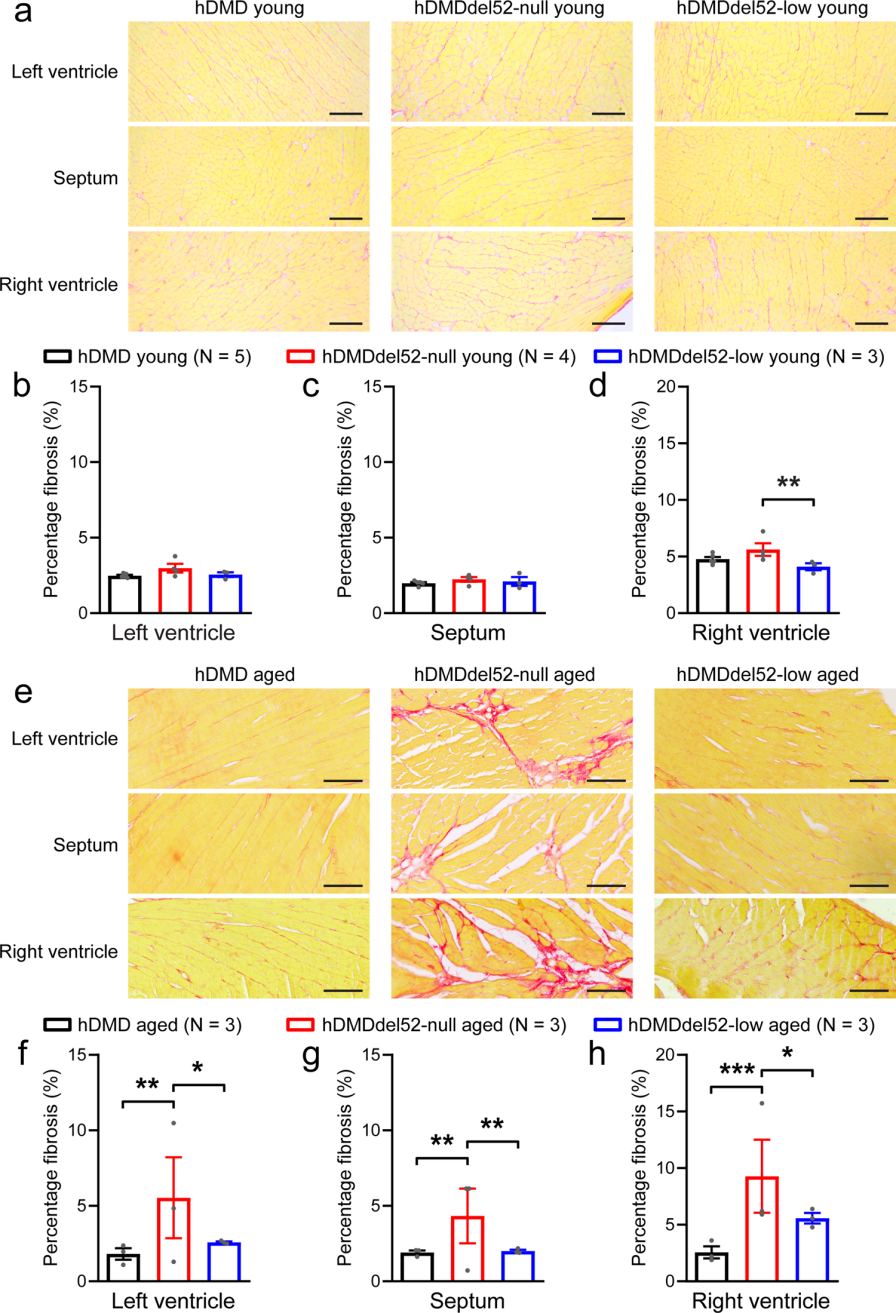
Enhanced myocardial fibrosis in aged mice in the absence of dystrophin, rescued in low dystrophin-expressing hearts. Picro Sirius Red staining was used to visualise and quantify myocardial fibrosis in hDMD, hDMDdel52-null, and hDMDdel52-low hearts. (**a**) Representative images used for analysis in young hearts. Average myocardial fibrotic area in left-ventricular (**b**), interventricular septal (**c**), and right-ventricular (**d**) myocardium measured in young hDMD (2.4 ± 0.2 months), hDMDdel52-null (3.0 ± 0.2 months), and hDMDdel52-low (3.2 ± 0.1 months) hearts. (**e**) Representative images used for analysis in aged hDMD (10.5 ± 0.9 months), hDMDdel52-null (11.2 ± 0.8 months), and hDMDdel52-low (10.7 ± 1.2 months) hearts. Average fibrotic area in left-ventricular (**f**), interventricular septal (**g**), and right-ventricular (**h**) myocardium. Scale bars: 100 µm, N represents the number of animals used for quantification. *P < 0.05, **P < 0.01, ***P < 0.001 (nested one-way ANOVA with Tukey’s post-hoc test).

We next quantified cardiac fibrosis levels by Picro Sirius Red staining in young and aged hDMD, hDMDdel52-null, and hDMDdel52-low hearts in order to assess the effect of different dystrophin expression levels on cardiac structural properties. (Fig. 2a,e). Fibrotic area was quantified as a percentage of total tissue in the left ventricular free wall (LV), interventricular septum (IVS), and right ventricular free wall (RV). No differences in fibrotic area were observed when comparing young hDMD and hDMDdel52-null hearts (Fig. 2b–d). However, more fibrosis was observed in the RV of young hDMDdel52-null hearts as compared to hDMDdel52-low (Fig. 2d). In aged mice, the amount of fibrosis in the LV, IVS, and RV was significantly higher in hDMDdel52-null hearts as compared to hDMD (Fig. 2f–h). In contrast, aged hDMDdel52-low mice showed similar fibrosis levels as hDMD, indicating that low levels of dystrophin were sufficient to prevent age-dependent fibrosis formation.

### Low dystrophin expression prevents abnormalities in AP upstroke and duration

**Figure 3:**
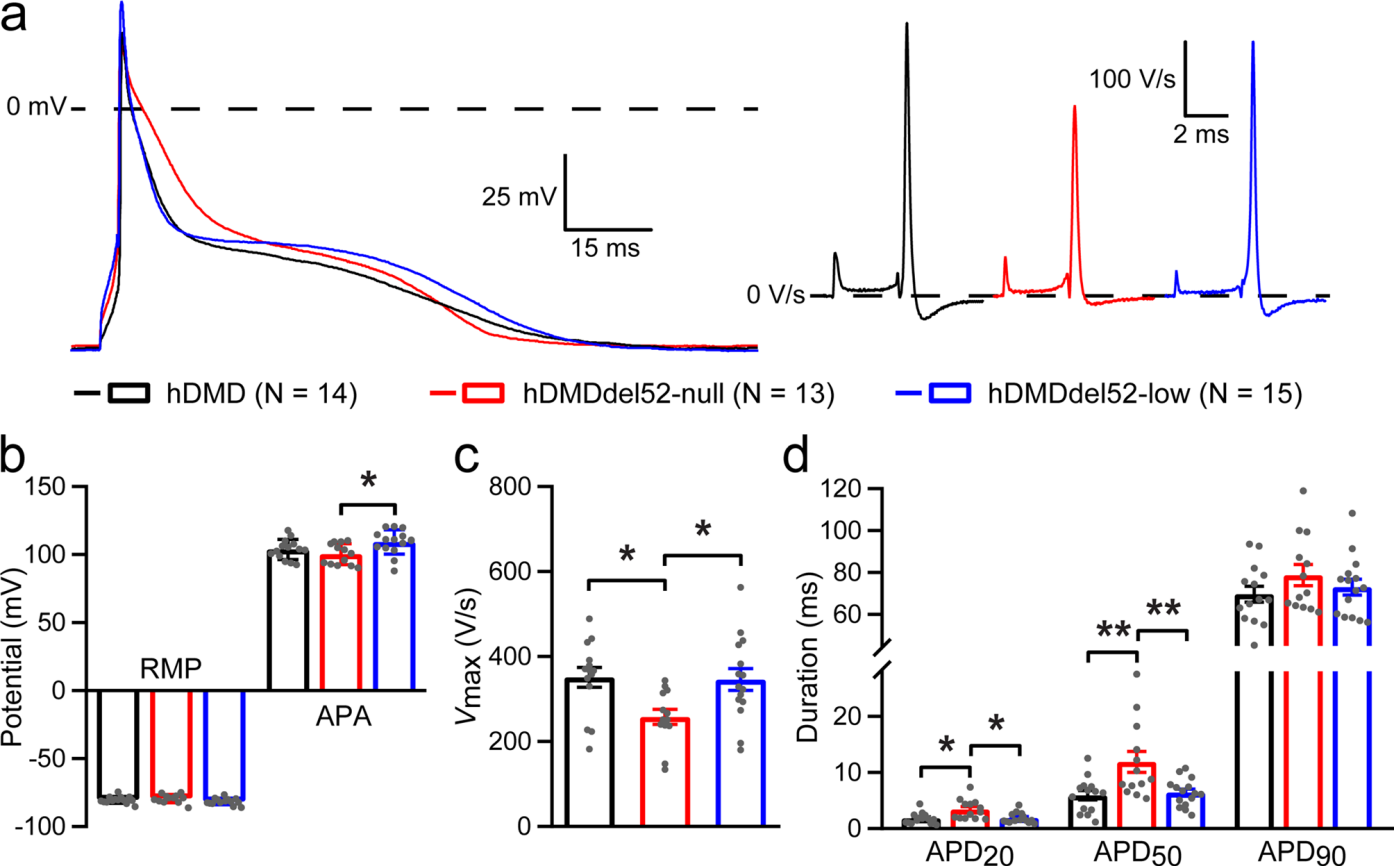
Single-cell action potential (AP) measurements reveal reduced action potential upstroke velocity and action potential duration prolongation upon loss of dystrophin, which is prevented by low dystrophin levels. (**a**) Typical examples of APs triggered at 2 Hz (left panel) and first derivative (d*V*/dt) of the AP upstroke (right panel) obtained in left ventricular cardiomyocytes from young hDMD (2.53 ± 0.03 months), hDMDdel52-null (2.37 ± 0.03 months), and hDMDdel52-low (2.43 ± 0.09 months) mice. (**b**) Average resting membrane potential (RMP) and AP amplitude (APA). (**c**) Average maximal AP upstroke velocity (*V*_max_). (**d**) Average AP duration at 20, 50, and 90% of repolarisation (APD_20_, APD_50_, and APD_90_ respectively). N represents the number of cardiomyocytes measured. Cardiomyocytes were isolated from 3 hDMD, 2 hDMDdel52-null, and 3 hDMDdel52-low mice. *P < 0.05, **P < 0.01 (one-way ANOVA with Tukey’s post-hoc test, Kruskal–Wallis with Dunn’s post-hoc test for APD_20_).

Following our observation that dystrophin deficiency in hDMDdel52-null mice resulted in ECG abnormalities at young age prior to the development of overt cardiac structural alterations, we next investigated the underlying cellular electrophysiological effects in single LV cardiomyocytes from young mice. Since ECG measurements revealed abnormalities on QRS duration and QT(c) interval, measures of ventricular activation and repolarisation respectively, we assessed cellular depolarisation and repolarisation by AP analysis. AP measurements at 2-Hz stimulation (Fig. 3a) revealed no differences in resting membrane potential (RMP) between the 3 groups, but a slight but significantly higher AP amplitude (APA) in hDMDdel52-low cardiomyocytes as compared to hDMDdel52-null (Fig. 3b). Maximal upstroke velocity (*V*_max_), which reflects cardiomyocyte excitability due to *I*_Na_, was significantly lower in hDMDdel52-null cardiomyocytes than in hDMD, but was restored to hDMD levels by expression of ~ 5% dystrophin in hDMDdel52-low cardiomyocytes (Fig. 3c). The impact of different dystrophin levels on cardiomyocyte repolarisation was assessed by measuring AP duration at 20, 50, and 90% of repolarisation (APD_20_, APD_50_, and APD_90_ respectively; Fig. 3d). A significant increase of both APD_20_ and APD_50_ was observed upon loss of dystrophin in hDMDdel52-null cardiomyocytes as compared to hDMD, which was prevented by expression of low dystrophin levels in hDMDdel52-low cells. In contrast, dystrophin expression levels did not significantly affect APD_90_. These results show that absence of dystrophin directly affects cardiomyocyte depolarisation and repolarisation prior to the development of cardiac structural alterations, which is prevented by the presence of low levels of dystrophin.

### Reduced *I*_Na_ in hDMDdel52-null is prevented by low dystrophin expression

**Figure 4:**
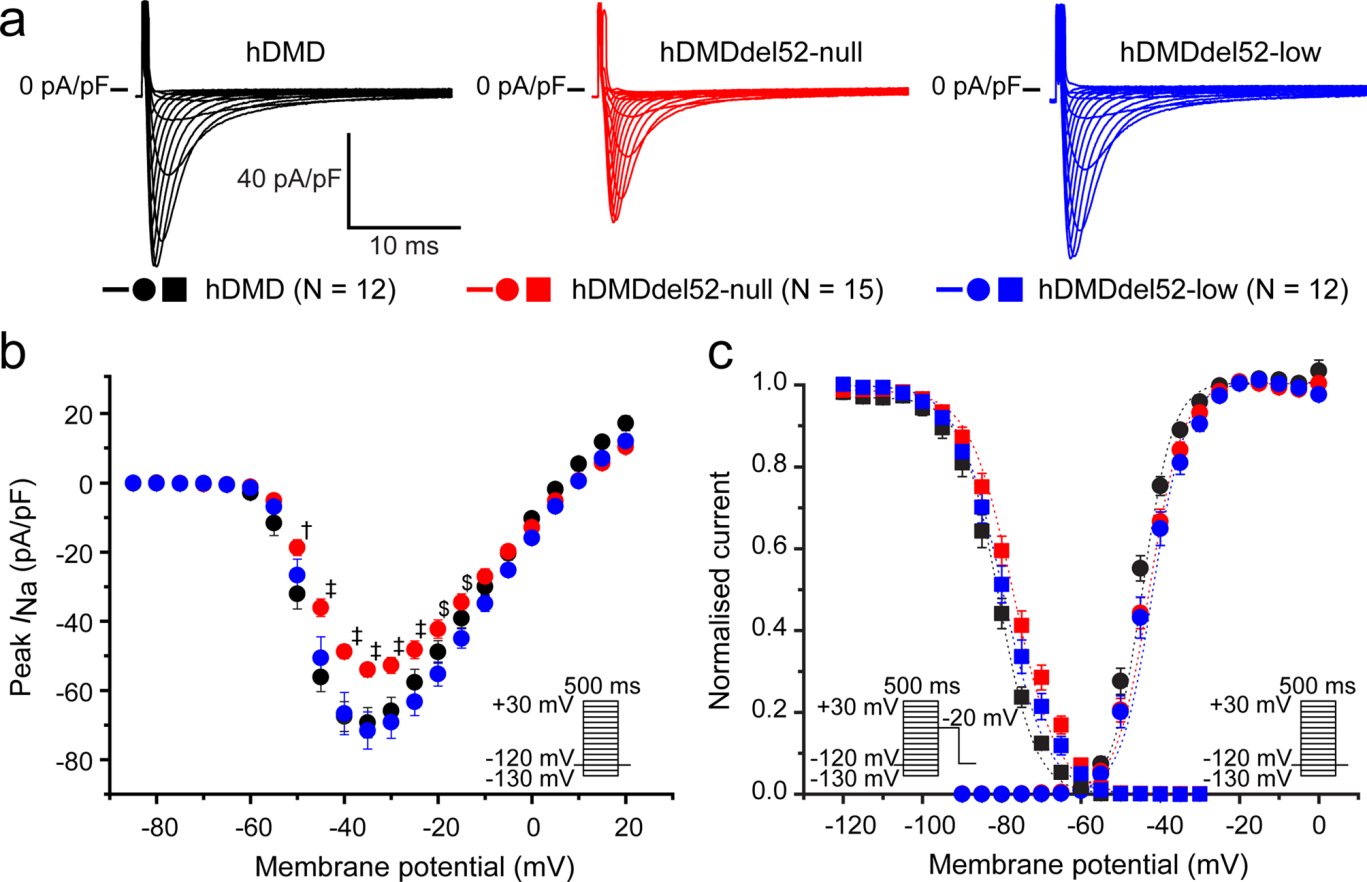
Sodium current (*I*_Na_) is reduced in dystrophin-null cardiomyocytes, which is prevented by low dystrophin levels. (**a**) Representative peak *I*_Na_ traces recorded in freshly isolated left ventricular cardiomyocytes from young hDMD (2.53 ± 0.03 months), hDMDdel52-null (2.40 ± 0.04 months), and hDMDdel52-low (2.43 ± 0.09 months). (**b**) Average *I*_Na_ current–voltage relationship, inset shows voltage-clamp protocol. (**c**) Voltage dependence of activation (circles) and inactivation (squares), dotted lines are Boltzmann fits. Curve of inactivation was shifted in hDMDdel52-null (see Table 1). Inset shows voltage-clamp protocol, N represents the number of cardiomyocytes measured. Cardiomyocytes were isolated from 3 hDMD, 4 hDMDdel52-null, and 3 hDMDdel52-low mice. † *P* < 0.05 for hDMDdel52-null vs. hDMD, ‡ *P* < 0.05 hDMDdel52-null vs. hDMD and hDMDdel52-low, $ *P* < 0.05 hDMDdel52-null vs hDMDdel52-low (two-way repeated measures ANOVA with Holm-Sidak post-hoc test).

We finally investigated whether the observed decrease in *V*_max_ in cardiomyocytes from young hDMDdel52-null mice is driven by changes in Na^+^ channel density or gating properties secondary to absence of dystrophin. Therefore, *I*_Na_ was measured in isolated LV cardiomyocytes from young mice by stepping to a potential ranging from − 130 mV to + 30 mV from a holding potential of − 120 mV (Fig. 4a). Average *I*_Na_ density per voltage step is presented in the *I*–*V* plot (Fig. 4b). Compared to hDMD cardiomyocytes, *I*_Na_ was significantly smaller in the absence of dystrophin in hDMDdel52-null cardiomyocytes, while low dystrophin levels in hDMDdel52-low were sufficient to normalise *I*_Na_ (Table 1, Fig. 4b). Voltage dependence of Na_V_1.5 activation, assessed by the potential at which half of the channels are opened (*V*_1/2_ of activation) and the slope factor *k* did not differ between groups (Table 1, Fig. 4c). In contrast, voltage dependence of inactivation was significantly different among groups, with hDMDdel52-null cardiomyocytes displaying a more positive potential at which half of the Na_V_1.5 channels were inactivated (*V*_1/2_ of inactivation) and a more negative slope factor *k* of inactivation as compared to hDMD cells; the *V*_1/2_ of inactivation was partially restored to hDMD levels in hDMDdel52-low cardiomyocytes (Table 1, Fig. 4c). Overall, this data indicates that absence of dystrophin in hDMDdel52-null leads to loss of *I*_Na_ and a positive shift in Na_V_1.5 voltage dependence of inactivation, which was prevented by low expression levels of dystrophin in hDMDdel52-low cardiomyocytes.

**Table 1:** Current density and gating characteristics of the sodium current (*I*_Na_). Half-voltage (*V*_1/2_) and slope factor (*k*) of the (in)activation curves were calculated using a Boltzmann fit. N represents the number of cardiomyocytes measured. Cardiomyocytes were isolated from 3 hDMD, 4 hDMDdel52-null, and 3 hDMDdel52-low mice. NS: no significant differences between groups, ^*a*^* P* < 0.05 vs hDMD and hDMDdel52-low, ^*b*^* P* < 0.05 vs hDMD, ^*c*^* P* < 0.0001 vs hDMD, ^*d*^* P* < 0.001 vs hDMD (one-way ANOVA with Tukey’s post-hoc test).

Parameter	hDMD (N = 12)	hDMDdel52-null (N = 15)	hDMDdel52-low (N = 12)	*P* value
Maximal peak *I*_Na_ (pA/pF)	− 72.05 ± 4.92	− 55.07 ± 2.49^*a*^	− 70.59 ± 5.66	< 0.05
*V*_1/2_ activation (mV)	− 45.05 ± 0.64	− 43.03 ± 0.74	− 42.71 ± 1.02	NS
*k* activation (mV)	4.35 ± 0.15	4.49 ± 0.09	4.64 ± 0.15	NS
*V*_1/2_ inactivation (mV)	− 80.90 ± 0.89	− 76.61 ± 1.09^*b*^	− 78.50 ± 1.24	< 0.05
*k* inactivation (mV)	− 5.37 ± 0.16	− 6.62 ± 0.18^*c*^	− 6.37 ± 0.15^*d*^	< 0.0001

## Discussion

We here demonstrate that dystrophin deficiency in mice leads to cardiac electrophysiological abnormalities from a young age onwards, prior to the development of myocardial fibrosis. Through patch clamp analyses, we show that reduced *I*_Na_ contributes to cardiac conduction slowing in young dystrophin-deficient mice. Furthermore, our results demonstrate that restoration of low levels of dystrophin (~ 5%) is sufficient to prevent both *I*_Na_ remodelling during the early disease phase, as well as cardiac fibrosis in late stages.

Cardiac involvement is well documented in patients suffering from DMD, including structural alterations such as fibrosis and cardiomyopathy, as well as electrical disturbances and arrhythmias. High heart rate^32–36^ and altered atrial and/or nodal conduction^35–37^, reflected by shortened PR duration, have been observed in a substantial fraction of DMD patients. In addition, altered ventricular activation^32,35–39^ and repolarisation^32–34^, detected by prolongation and/or abnormal morphology of the QRS complex, and prolonged QT(c) duration respectively, has also been reported in some but not all DMD patients. Both repolarisation abnormalities and ventricular conduction slowing are well-established facilitators of cardiac arrhythmias. Indeed, arrhythmias are widely observed in DMD patients, with around 60% of adolescent patients exhibiting atrial- and/or ventricular premature beats^40,41^. Additionally, cardiomyopathy and progressive myocardial fibrosis is commonly found in DMD patients^42,43^. ECG disturbances have been reported prior to the development of cardiomyopathy^7,9^, suggesting that cardiac electrical dysfunction precedes structural abnormalities in DMD patients. Accordingly, we here show cardiac activation and repolarisation disturbances independent of myocardial fibrosis in young hDMDdel52-null mice. As these electrophysiological effects were exacerbated in aged animals, where myocardial fibrosis was increased, dystrophin deficiency is potentially pro-arrhythmic through various electrophysiological and structural alterations, which may act in concert. Although the fibrosis assessment was performed in relatively few hearts from aged animals due to limited availability, the differences in fibrosis were both visually and quantitively striking. Activation and repolarisation disturbances have also been described in multiple other DMD mouse models, including QRS^19,20,44–46^ and/or QT(c)^44–46^ prolongation, but were not present in other DMD mouse models^19,47^. Also, while increased heart rate^10,44,45^ and PR shortening^20,44–47^ are commonly described in DMD mouse models, these parameters were not affected in hDMDdel52-null mice. Hence, DMD mouse models display a range of cardiac phenotypes. This variability in the cardiac consequences of dystrophin absence could potentially be explained by genetic background, differences in dystrophin ablation, and type of anaesthetic used during ECG measurements as well as depth of anaesthesia. While isoflurane has been reported to potentially affect PR, QT, and QRS interval^48^, low concentrations were used in all experimental groups. Moreover, the conduction- and repolarisation alterations measured by ECG were in line with patch clamp recordings in isolated cardiomyocytes, confirming the in vivo phenotype. As such, our observations in young hDMDdel52-null animals correspond to findings in young DMD patients, who display ECG abnormalities in the absence of overt structural defects^8,9^.

The hDMDdel52-null mouse model expresses human dystrophin with a partial deletion in exon 52 (first 25 bp)^49^, located in a hotspot where mutations are found in most DMD patients^50^, and therefore closely represents the mode of dystrophin loss in patients. In addition, the models used here also carry a nonsense mutation in exon 23 of the mouse *Dmd* gene, thus abolishing production of mouse dystrophin. Of note, while revertant fibres spontaneously regaining dystrophin expression are relatively frequent in skeletal muscle, also in these models^30^, these are observed at an extremely low rate and are therefore considered not relevant in cardiac tissue^51^. Binding of syntrophin to dystrophin is essential for proper formation and function of the dystrophin-glycoprotein complex. The amino acid sequence of human and murine dystrophin and syntrophin show a high level of similarity (> 90%). Crucially, the spectrin-like repeats in the carboxy-terminal region of dystrophin, which are known syntrophin-binding sites, are identical in human and murine dystrophin. Additional predicted syntrophin binding sites are also highly similar or identical between both species. Hence, human dystrophin and murine syntrophins are extremely likely to interact^52^. Indeed, endogenous (murine) syntrophin showed clear co-localization with human dystrophin in quadriceps tissue of hDMD mice (Supplemental Fig. 1).

The interaction of dystrophin with Na_V_1.5 occurs via syntrophin, with PDZ-domain of the latter binding to the SIV motif located in the C-terminus of Na_V_1.5^19^. Disruption of this interaction in *Scn5a*-ΔSIV mice was previously shown to result in loss of Na_V_1.5 and *I*_Na_ specifically at the lateral membrane of cardiomyocytes, in addition to ECG abnormalities and conduction slowing^53^. In accordance with previous studies^19,20,23^, we here show loss of *I*_Na_ upon dystrophin absence, leading to conduction slowing and potentially to arrhythmias. In our current study, *I*_Na_ loss was combined with a positive shift in the *V*_1/2_ of Na_V_1.5 inactivation, which was also found in a previous study using a different DMD mouse model^23^. This shift in inactivation theoretically results in increased Na_V_1.5 availability, possibly compensating for the loss of *I*_Na_. Nevertheless, a decreased AP *V*_max_ was still observed in hDMDdel52-null cardiomyocytes, demonstrating that this shift in *V*_1/2_ of Na_V_1.5 inactivation was not sufficient to negate the loss of *I*_Na_. Crucially, *I*_Na_ loss occurred prior to the development of myocardial fibrosis, and is therefore independent of structural dysfunction. Of note, cardiac structural abnormalities including increased fibrosis have also been reported in the setting of Na_V_1.5 dysfunction^54–56^, suggesting that loss of Na_V_1.5 can cause cardiac structural remodelling. Therefore, it remains to be established whether fibrosis formation in DMD is driven by primary loss of dystrophin, or (additionally) mediated by secondary loss of Na_V_1.5/*I*_Na_.

Interactions between dystrophin (either direct or indirect via syntrophin or dystroglycans) and various ion channels may play an essential role in the regulation and localisation of the latter at the cell membrane^57^. Dystrophin is also a microtubule-associated protein, and loss of dystrophin has been shown to result in microtubule disorganisation in skeletal muscle which was prevented by expression of a mini-dystrophin in *mdx* mice^58,59^. Hence, loss of dystrophin may also negatively impact on microtubule-dependent trafficking of Na_V_1.5 to the membrane. We here show restoration of AP characteristics and *I*_Na_ in hDMDdel52-low cardiomyocytes, indicating that ~ 5% of normal dystrophin levels is sufficient to ensure the incorporation of Na_V_1.5 in the sarcolemma. The exact mechanisms by which a fraction of normal dystrophin restores *I*_Na_ however remains to be fully elucidated.

In line with our findings, previous studies demonstrated that the cardiac DMD phenotype could also be prevented by expression of human micro- and mini-dystrophins in *mdx* mouse models. Both ECG defects and cardiac fibrosis were prevented in *mdx* mice treated with an recombinant adeno-associated virus encoding a human micro-dystrophin^60^. Moreover, various studies employing transgenic *mdx* mouse lines expressing cardiac-specific mini-dystrophins also showed a largely prevented cardiac electrical, structural, and functional phenotype^44,46,61^*.* Other approaches to assess the impact of dystrophin rescue on cardiac function and structure include DMD mouse models expressing dystrophin in a mosaic, or heterogeneous manner. While female carrier mice expressing around 50% of the normal levels of dystrophin in a mosaic manner were unaffected^10^, low dystrophin levels were associated to an alleviated (cardiac) DMD-like phenotype in *mdx* and dystrophin/utrophin double-knockout mice expressing full-length murine dystrophin in a mosaic manner^12,62^. In contrast, low levels (~ 3.3%) of near full-length murine dystrophin in all muscle fibres in the *mdx*^*3cv*^ mouse model, only resulted in partially alleviated ECG defects and hemodynamics, while cardiac fibrosis was not prevented^45^. This discrepancy in the effect of low expression of dystrophin in DMD mouse models may be explained by differences in expression levels, but potential differences in human and murine dystrophin function may also be a contributing factor. Therefore, the findings in the hDMDdel52-low model are relevant for understanding how low expression levels of human dystrophin modulates cardiac electrophysiology and structure. Novel DMD therapy strategies, including gene addition therapy and antisense oligonucleotide-mediated exon skipping so far result in partial restoration of dystrophin expression^63^. Current challenges in the development of antisense oligonucleotide-mediated exon skipping include targeting cardiac tissue, as efficiency is currently low. Crucially, our current study demonstrates that low dystrophin expression in most fibres is sufficient to prevent cardiac involvement in a murine DMD model. Therefore, achieving low-level cardiac dystrophin expression can be regarded as a potential therapeutic goal in DMD patients. While improvement of the cardiac DMD phenotype is observed upon mosaic dystrophin expression in mouse models^12,62^, uniform dystrophin expression would be favourable in order to prevent electrical heterogeneity.

In conclusion, we show that the hDMDdel52-null mouse model exhibits ventricular activation and repolarisation defects from a young age onwards, preceding the development of structural abnormalities in aged mice. These defects were absent in hDMDdel52-low mice, demonstrating that low dystrophin levels are sufficient to prevent both the cardiac electrophysiological and age-dependent structural DMD phenotype. Our findings are of potential relevance for the development of novel therapeutic strategies aimed at restoring dystrophin expression in DMD.

## Methods

### Generation and breeding of mice

All mouse lines were generated and bred at the LUMC facility as described previously^30^. Animals were transported to the Amsterdam UMC animal facility and acclimatised for 2 weeks before further handling. Mice were housed in individually ventilated cages in rooms with a 12-h light/dark cycle at a temperature of 20 ± 2 °C and 40–70% humidity. Ad libitum access to chow (Teklad #T.2916.12.F2 with fenbendazole) and water was provided to mice. All methods were carried out in accordance with relevant guidelines (including ARRIVE guidelines) and the study design and all animal handling and experiments were approved by governmental and Institutional Animal Care and Use Committees of the University of Amsterdam (license 18–4986) and the Leiden University Medical Center (license 14,180). Transgenic mice from the previously described B6.DBA2.129-hDMD^tg/tg^/LUMC*B10-Dmd^*mdx*^/J line (hDMD), expressing no functional murine dystrophin and normal levels of functional human dystrophin were used as a healthy control^31^. Additionally, we employed the del52hDMD/*mdx#*35 (hDMDdel52-null), and del52hDMD/*mdx#37* (hDMDdel52-low) mouse lines^30,49^. As a partial deletion of exon 52 (first 25 bp) was introduced in the human *DMD* gene in these transgenic mice, human dystrophin expression was abolished in hDMDdel52-null mice^49^. However, trace levels of dystrophin (~ 5%) were found in hDMDdel52-low, with most myofibers expressing low levels of human dystrophin^30,31,64^. Of note, all models used also carried a nonsense mutation in exon 23 of the mouse *Dmd* gene, and hence also lacked murine dystrophin. For each mouse line, young (age 2–3 months) and aged (age 6–13 months) male mice were used. Average ages of mice used in the various experiments were not significantly different between experimental groups, and are listed in the respective figure legends.

### In vivo surface electrocardiogram measurements

Mice were anesthetized using isoflurane inhalation (0.8–1.2% volume in oxygen), and surface ECGs were recorded using the Powerlab acquisition system and analysed using LabChart 8 Pro software (ADInstruments, Sydney, Australia). Care was taken to ensure that the ECG measurements were performed using low isoflurane concentrations; this was carefully monitored by regularly checking the effect on breathing frequency. Electrodes were placed in the right and left armpit, and the left groin, while a reference electrode was placed in the right groin. From these leads, a standard 3-lead ECG was generated as described previously^65^. Since lead II had the best quality signal overall, this lead was used for analysis. QTc was calculated according to the method from Mitchell et al.^66^.

### Fibrosis quantification

Cardiac fibrosis was assessed in young and aged mice. The hearts from 5 young hDMD, 3 hDMDdel52-null, and 4 hDMDdel52-low mice were acquired, processed and stained with Sirius Red as described elsewhere^67^. For assessment of cardiac fibrosis in aged mice, hearts of 3 aged hDMD, 3 hDMDdel52-null, and 3 hDMDdel52-low mice were fixed overnight in 4% paraformaldehyde and transferred to 70% ethanol until embedding in paraffin. Paraffin sections of 5 µm thickness were dewaxed and hydrated before 60 min incubation in Picro Sirius Red Stain solution. Subsequently, sections were differentiated for 2 min under continuously moving in 0.01 N HCl, dehydrated, and mounted in entellan (Merck, Darmstadt, Germany). Per heart, 35 pictures from 5 sections were obtained using a light microscope (Leica DM5000, Wetzlar, DE and Keyence BZ-X700, Osaka, Japan) at 20 × magnification (i.e. 15 pictures in the left ventricular free wall, 10 in the septum, and 10 in the right ventricular free wall). Perivascular fibrosis was manually removed from the pictures. Sirius Red positive pixels and total tissue area were independently determined two blinded researchers by manually adjusting threshold settings in ImageJ (Version 1.52a). Sirius Red-positive area was calculated in each picture as a percentage of the total tissue area, the average of the Sirius Red-positive area quantified by both researchers is reported.

### Single cell preparation

After ECG analysis, mice were sacrificed by cervical dislocation and the heart was isolated. Excised hearts were retrogradely perfused in a Langendorff system with oxygenated modified Tyrode’s solution (37 °C) containing (in mM): NaCl 140, KCl 5.4, CaCl_2_ 1.8, MgCl_2_ 1.0, glucose 5.5, and HEPES 5.0; pH 7.4 (NaOH). After 10 min, hearts were perfused for 10 min with nominally Ca^2^^+^-free modified Tyrode’s solution containing 0.01 mM CaCl_2_, supplemented with 10.7 mM creatine. Subsequently, the heart was digested using Liberase TM (0.032 mg/mL; Roche, Basel, Switzerland) and elastase (1.6 U/mL; SERVA Electrophoresis GmbH, Heidelberg, Germany) in nominally Ca^2^^+^-free modified Tyrode’s perfusion solution for around 10 min. The heart was then placed in nominally Ca^2^^+^-free modified Tyrode’s perfusion solution containing 1% bovine serum albumin (Sigma-Aldrich, St. Louis, MI, USA). Atria and right ventricular free wall were removed, and the left ventricle was mechanically dissociated to obtain single cardiomyocytes. The cells were then washed with nominally Ca^2^^+^-free modified Tyrode’s solution, followed by washing steps with modified Tyrode’s containing 0.9 mM CaCl_2_ and 1.8 mM CaCl_2_ respectively. Cells were stored in modified Tyrode’s solution containing 1.8 mM CaCl_2_ at room temperature until measurements were performed.

### Patch-clamp data acquisition

Cellular electrophysiological measurements were performed using an Axopatch 200B amplifier (Molecular Devices Corporation, Sunnyvale, CA, USA). Borosilicate glass patch pipettes with a tip resistance of 2–2.5 MΩ were used for whole-cell *I*_Na_ and AP measurements. For *I*_Na_ measurements, cell membrane capacitance (*C*_m_) was determined by dividing the decay time constant of the capacitive transient in response to 5 mV hyperpolarizing steps from − 40 mV, by the series resistance (*R*_s_). Measurements were filtered at 5 kHz and digitized at 40 kHz.

### Voltage-clamp experiments to measure *I*_Na_

*I*_Na_ was measured using the ruptured patch-clamp technique. Glass pipettes were filled with a solution containing (in mM): NaCl 3.0, CsCl 133, MgCl_2_ 2.0, Na_2_ATP 2.0, TEACl 2.0, EGTA 10.0, HEPES 5.0; pH 7.2 (CsOH). Measurements were performed in a bath solution containing (in mM): NaCl 7.0, CsCl 133, CaCl_2_ 1.8, MgCl_2_ 1.2, glucose 11.0, HEPES 5.0, nifedipine 0.005; pH 7.4 (CsOH). Series resistance and cell membrane capacitance were compensated for 80–90%. Peak *I*_Na_ was measured at room temperature (21 °C) from a holding potential of − 120 mV following steps of 5 mV from − 130 mV to + 30 mV, with a cycle length of 5 s. *I*_Na_ was defined as the difference between peak current and steady-state current. *I*_Na_ density was calculated by dividing *I*_Na_ amplitude by cell membrane capacitance (*C*_m_). Potentials for peak *I*_Na_ recordings were not corrected for the estimated change in liquid junction potential. Na_V_1.5 voltage dependence of activation and steady-state inactivation curves were fitted with a Boltzmann equation ($y =(1 + exp\{(V-{V}_{1/2})/k\})-1$), where *V*_1/2_ is the voltage at half-maximal (in)activation, and *k* is the slope factor.

### Action potential measurements

In single mouse left ventricular cardiomyocytes, APs were measured at 36 °C using a modified Tyrode’s solution containing (in mM): NaCl 140, CaCl_2_ 1.8, MgCl_2_ 1, KCl 5.4, HEPES 5; pH 7.4 (NaOH) as bath solution. Pipettes were filled with (in mM): K-gluconate 125, KCl 20.0, NaCl 5.0, amphotericin-B 0.44, HEPES 10, pH 7.2 (KOH). APs were elicited at 2 Hz by 2-ms, ≈1.2 × threshold current pulses through the patch pipette. We analysed resting membrane potential (RMP), AP amplitude (APA), maximal AP upstroke velocity (*V*_max_) and AP duration (APD) at 20%, 50% and 90% repolarisation (APD_20_, APD_50_ and APD_90_, respectively). Data from 10 consecutive APs were averaged and potentials were corrected for the calculated liquid junction potential of 15 mV^68^.

### Statistical analyses

Shapiro–Wilk normality tests, and accordingly one-way ANOVA with a Tukey’s multiple comparisons post-hoc analyses for parametric data, or Kruskal–Wallis tests with a Dunn’s multiple comparisons test for non-parametric data, were performed with GraphPad Prism software (version 8, GraphPad Software, San Diego, CA, USA). For cardiac fibrosis data, a nested one-way ANOVA with a Tukey’s multiple comparison test was performed in GraphPad Prism, with the observed fibrotic area per section as input. Statistical significance for differences in *I*–*V* curves were determined by performing a two-way repeated measures ANOVA followed by a Holm-Sidak post hoc analysis in SigmaStat software (Version 3.5, Systat Software Inc., San Jose, CA, USA). For all tests, a *P*-value lower than 0.05 was considered statistically significant. Mean data is presented ± Standard Error of Mean (SEM).

## Supplementary Information

Supplementary Information.

## Data Availability

The data that support the findings of this study are available from the corresponding author upon reasonable request.
