# Supplementary material for: Low human dystrophin levels prevent cardiac electrophysiological and structural remodelling in a Duchenne mouse model
Source: Sci Rep. 2021 May 7;11:9779. doi: 10.1038/s41598-021-89208-1 (PMC8105358; doi:10.1038/s41598-021-89208-1)
Supplement: Supplementary file 1 — Supplementary Information. [file 41598_2021_89208_MOESM1_ESM.docx]

**Low human dystrophin levels prevent cardiac electrophysiological and structural remodelling in a Duchenne mouse model**

**-SUPPLEMENTAL MATERIALS-**

**Authors:**

Gerard A. Marchal^1*^, Maaike van Putten^2^, Arie O. Verkerk^1,3^, Simona Casini^1^, Kayleigh Putker^2^, Shirley C.M, van Amersfoorth^1^, Annemieke Aartsma-Rus^2^, Elisabeth M. Lodder^1^, Carol Ann Remme^1^

1: Amsterdam UMC (location AMC), Department of Experimental Cardiology, Meibergdreef 9, 1005 AZ Amsterdam, The Netherlands

2: Leiden University Medical Center, Department of Human Genetics, Albinusdreef 2, 2333 ZA Leiden, The Netherlands

3: Amsterdam UMC (location AMC), Department of Medical Biology, Meibergdreef 9, 1005 AZ Amsterdam, The Netherlands

* Corresponding author

**Correspondence to:**

Gerard A Marchal: g.a.marchal@amsterdamumc.nl; +31 20 56 63266

# Supplemental Figures


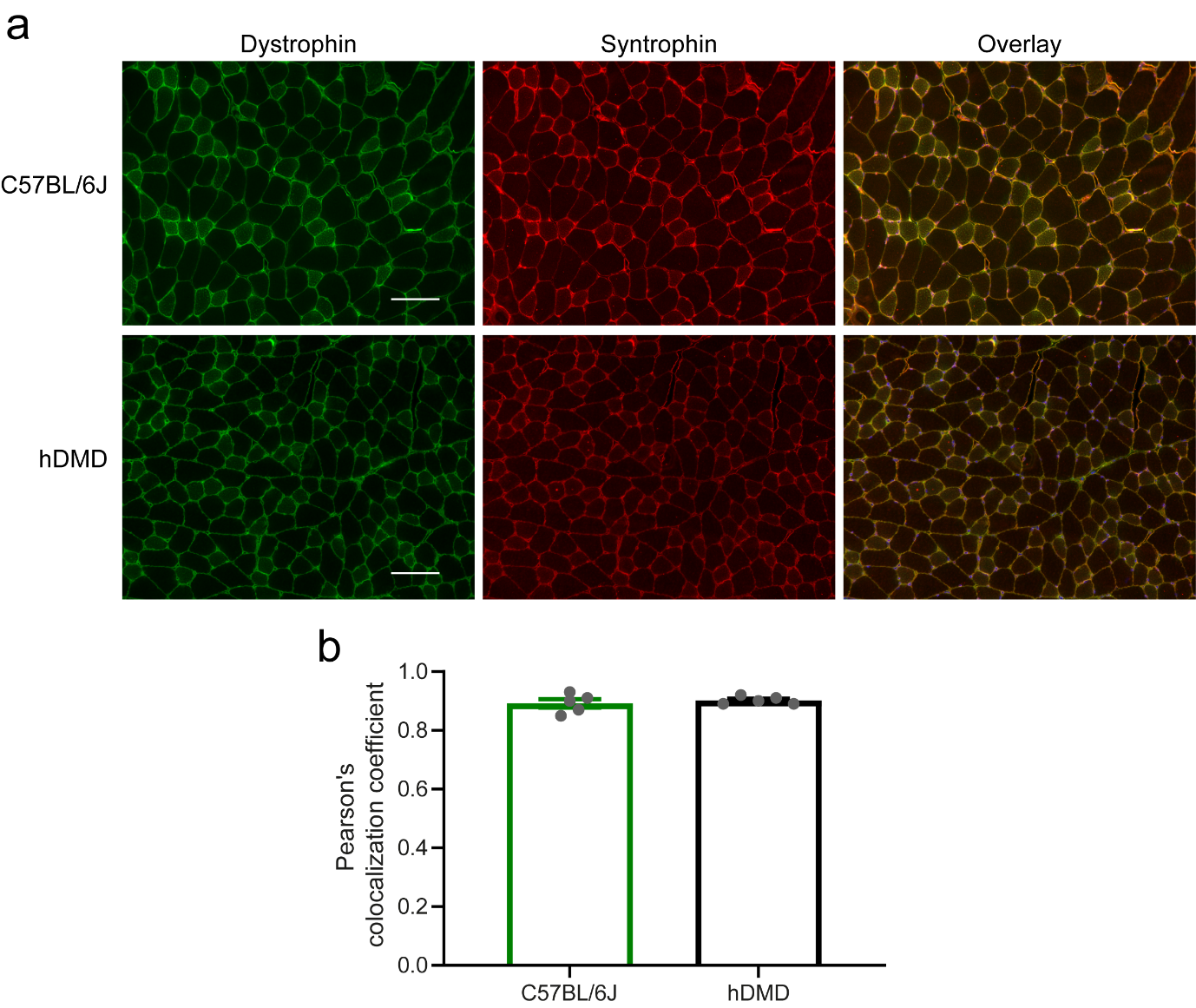


**Supplemental Figure 1:** (**a**) Immunohistochemistry staining visualising dystrophin and syntrophin in quadriceps muscle tissue of C57BL/6J (wild type) and hDMD (expressing 100% human dystrophin) Scale bars: 100 μm. (**b**) Quantification of dystrophin and syntrophin colocalization reveals a similar degree of colocalization in C57BL/6J and hDMD. N = 5 ROIs per group (Unpaired Student’s t-test).

# Supplemental Methods

## Immunohistochemistry and colocalization analysis in murine quadriceps

Quadriceps of C57BL/6J mice (expressing 100% murine dystrophin) and hDMD mice (expressing 100% human dystrophin, 0% murine dystrophin were snap-frozen and sectioned at 8 µm. Sections were incubated with primary antibodies against dystrophin (Santa Cruz sc-73592; 1:50 in blocking buffer) and syntrophin (Thermo PA5-77702; 1:100 in blocking buffer) overnight at 4 °C. Next, the sections were washed 3 times with PBS, and subsequently incubated 1 hour at room temperature with the appropriate secondary antibodies (anti-Mouse conjugated with Alexa Fluor 488 (Thermo A-11001, 1:1000 in blocking buffer), and anti-Rabbit conjugated with Alexa Fluor 594 (Thermo A-11012, 1:1000 in blocking buffer). After washing coverslips were mounted in Prolong Gold containing DAPI (Thermo P36931). Imaging was performed on a Keyence BZ-X700 microscope.

Next, confocal microscopic images were used to quantify colocalization of dystrophin and syntrophin by assessing overlap of the two fluorescent signals. Images of the individual fluorescent channels were loaded in Fiji-ImageJ (Version 1.53) and transformed to 8-bit grayscale images. Next, background noise was removed, and five regions of interest (ROIs) were set per sample. Within these ROIs, colocalization of fluorescence between the two channels was assessed, presenting the degree of overlap by a Pearson’s colocalization coefficient.
